# Exploring Primary Healthcare Students and Their Mentors’ Awareness of Mentorship and Clinical Governance as Part of a Local Continuing Professional Development (CPD) Program: Findings of a Quantitative Survey

**DOI:** 10.3390/healthcare7040113

**Published:** 2019-10-02

**Authors:** Robert McSherry, Michael Snowden

**Affiliations:** 1 Nursing and Practice Development, National Health Service Calderdale Clinical Commissioning Group, Halifax HX3 5AX, UK; robert.mc1527@gmail.com; 2 School of Human and Health Sciences, University of Huddersfield, Huddersfield HD1 3DH, UK

**Keywords:** mentee, mentor, patient safety, quality, individual, personal, characteristics, awareness of mentors

## Abstract

*Introduction:* Previous research exploring the benefits of mentoring and the place of clinical governance in enhancing care delivery illustrated an unexplored synonymous relationship between mentors and mentees (students at undergraduate and postgraduate levels) and its potential impact on patient safety and quality of care. The significance of the research was in recognizing the importance the role of the mentor can play in raising awareness of patient safety and clinical governance principles and processes in the primary healthcare setting. *Aims:* Building on this preliminary research, this research aimed to explore primary healthcare workers and their mentor’s awareness of mentorship and clinical governance as part of a local Continuing Professional Development (CPD) program. Furthermore, it aimed to establish any relationship between the mentors, the mentee, and their awareness and application of clinical governance in the primary healthcare setting. *Methodology*: A quantitative research design using a survey was adopted. *Data Collection Instrument:* The researchers integrated previously validated questionnaires incorporating a Mentor Potential Scale, the Dimensions of Mentoring, and a Clinical Governance Awareness Questionnaire into a new questionnaire. This was called “Mentorship and Clinical Governance Awareness”. *Sample:* Convenience sample surveys were posted to complete and return to 480 primary healthcare workers undertaking post graduate study. *Findings*: A total of 112 completed questionnaires were included for the analysis amounting to a 23% response rate. A principle component factor analysis combining part 1— the characteristics of an effective mentor and part 2—the personality characteristics of an effective mentor identified four primary characteristics. These are: (1) “A Facilitatory Adviser”, (2) “Critically Enabling Facilitator”, (3) “A Change Facilitator”, and 4) “An Approachable Facilitator”. These newly identified characterizations according to the primary healthcare workers significantly impacted on their awareness and application of clinical governance in primary healthcare practice. *Implications for primary healthcare practice and education:* The newly devised questionnaire can be used to gauge the effectiveness of mentors and mentoring and how the characteristics of the role can impact on mentee’s awareness and application of clinical governance. Healthcare manager’s, leaders, and educators should focus their attention on how these newly established characteristics of the mentor can influence clinical governance awareness and application in healthcare the future.

## 1. Introduction

Globally, patient safety is a growing concern for all health and social care services irrespective of location. Across the world, 10,000 individuals are treated safely everyday resulting in improvement in health and lifestyle. However, an unacceptable number of patients are harmed due to either human and/or mechanical errors/factors or both [1].

Healthcare workers should be commended for the quality of care and services they provide, this is despite the ongoing socio-economic, political, and professional challenges they face. The majority of healthcare workers want to, and do, provide excellent care and services to their patients and carers [2]. Ensuring the provision of safe, caring, and compassionate services play a pivotal role in ensuring sound patient and service outcomes. Unfortunately, health and care’s public and profession image for delivering safe, quality, compassionate care, and services has been tarnished [3,4,5]. This is because of a successive ongoing outcry from the media and the public, following numerous scandals, investigations, and inquires highlighting neglectful and harmful care. In the United Kingdom (UK) examples include the Public [6] and Independent [7] inquiries into the Mid Staffordshire National Health Service Foundation Trust, The Winterbourne Care Home scandal [8], and the Kirkup Inquiry into midwifery and neonatal care [9]. The UK is not alone in what appears to be an international trend where care and services seem to be compromised as illustrated by incidents in Australia [10], the United States of America [11], and Ireland [12].

To address these rising trends, healthcare professions advisory and regulatory bodies place due emphasis on the importance of patient safety, quality, and governance. This is imperative in ensuring the promotion of health, prevention of illness, and caring for the individual during a time of challenge and need throughout the life span. For example, the International Council for Nursing (ICN) [13] (p1) advocate the:

“Promotion of a safe environment, research, participation in shaping health policy and in patient and health systems management, and education”.

Furthernore, the ICN [13] and other regulatory bodies such as the Nursing and Midwifery Council (NMC), United Kingdom (UK) [14], Nursing and Midwifery Board of Australia (NMBA) [15], and the American Nurses Association (ANA) [16] all recognize the importance of ensuring patient safety, quality, and governance in healthcare practice. The quest for safety and quality are also advocated by other professional disciplines, such as medicine (General Medical Council (GMC) United Kingdom).

Some questions arising from such scandals and emerging trends showing a possible decline and erosion in the quality of care and services are as follows: How is it possible that such situations are continually occurring in the UK, Ireland, and the USA when quality systems and frameworks have been introduced to safeguard and protect both patients and the public? Are healthcare workers unaware of the importance of these quality systems and frameworks? Have they, and do they, receive any clinical governance education and training as part of either undergraduate or postgraduate education?

The originality and unique nature of this research paper is in illustrating and exploring the role and responsibilities of the mentee and mentors in promoting patient safety and sound governance principles in clinical practice. Furthermore, the research highlights the personal and individual characteristics of the mentor and how these are key in facilitating this to happen in healthcare practice. The research explores Darwin’s [17] and Darling’s [18] seminal works on mentoring and how these can influence contemporary practice in the future.

### 1.1. Literature Review

A review of the literature highlighted an emerging relationship between clinical governance and mentoring and the characteristics of the mentor in supporting the application of clinical governance. These are explored in more detail below.

#### 1.1.1. Evolution of Clinical Governance and the Relationship with Mentoring

Clinical governance was introduced within the UK National Health Service in 1997 with the sole purpose of improving patient safety, quality care, and services by reducing risks and avoiding errors [19,20]. Clinical governance is defined as:

“A framework through which NHS organizations are accountable for continuously improving the quality of their services and safeguarding high standards of care by creating an environment in which excellence in clinical care will flourish”

[20].

From its origins, the term clinical governance has become the mantra for quality care and all things patient safety services across the world. This is because it is regarded as a whole system framework designed to minimize risks to patient and staff safety [19,20]. This is achieved by facilitating quality improvement(s) through raising professional’s awareness of their own accountability for excellence [19].

A research-based case study by Ellwood et al. [2], exploring the benefits of mentoring and the place of clinical governance in enhancing dental care, illustrated an unexplored synonymous relationship between mentors and mentees (students at undergraduate and postgraduate levels) and its potential impact on patient safety and quality. The significance of Snowden et al [21] and Ellwood et al.’s [2] work is in recognizing the importance the role of the mentor can play in raising awareness of patient safety and quality in practice. The findings demonstrated that mentoring is a potentially powerful instrument for improving patient safety and quality in several ways:

Firstly, a layer of support is provided that enables the mentee to access the knowledge, skills, experience, expertise, and wisdom of the mentor in developing their practice.

Secondly, the facilitatory process of the relationship can promote confidence, assertiveness, and also improves negotiation skills and the desire to encourage excellence, for example, where the mentor encourages their mentee to administer and manage medicine safely. This is achieved by developing cross-checking strategies for ensuring their own and the patients’ safety prior to, during, and post-administration of medication.

Thirdly, the reciprocity of the relationship provides the following. A safe, caring clinical learning and teaching environment where the mentor and mentee can mutually reflect upon real-time practice, events, situations, and experiences to provide optimal learning, development, and sharing between both parties, for example, when the mentor recognizes and praises their mentee for performing their role exceptionally well.

Finally, it is clear that mentoring has great potential to promote a proactive approach to both a personal and professional development by increasing both the mentor’s and mentee’s awareness of clinical governance and how it can be utilized to enhance practice through the holistic developmental process associated with mentoring. For example, reflecting upon clinical and non-clinical situations, incidents, and events where patient care is outstanding and/or where improvements are required.

#### 1.1.2. Characteristics of Mentoring in Supporting the Application of Clinical Governance

Darling’s [18] key characteristics of a mentor naturally lend themselves to support the application of clinical governance in several ways. By empowering the mentee to bounce off their ideas and experience akin to their situation, working events, during the provision, and/or after an episode of care with the mentor. This is essential in order to open their eyes, challenge, and problem-solve, through prodding and probing which will increase their confidence, competence, and capability in clinical nursing practice. Furthermore, the proactive learning and sharing from incidents, events, and scenarios within a clinical governance framework mirror the facilitative and enabling nature of the mentoring relationship. This is because fundamentally both clinical governance and mentoring are built on the principles of trust, honesty, openness, transparency, and having the ability to raise/escalate concerns without comeback and reprisal.

In relation to the above context, it is clear that mentors are deemed to be:

“Highly experienced custodians and advocates of safe, quality and compassionate care providing effective role modelling, guidance, supervision and support to less experienced and aspiring practitioners”

[2] (p. 386).

However, it is also important to acknowledge that mentoring is difficult to define [22,23,24]. This is because it is a socially constructed term, highly complex, multifaceted, and is context and/or professional/discipline-specific [24,25,26,27,28]. For example, business, medicine, nursing, dentistry, law, and education all recognize the importance that the mentoring role has on personal development and the development of others. However, the nature of the role is different within each context. For example, business mentors are akin to coaches, nursing and dental mentors are viewed as assessors of practice, and education mentors view the role as lined to professional development. It is this lack of clarity that generates confusion and undermine the potential influence of the mentor [25,26,27,28,29] within practice. It is this lack of clarity and conflict about the role with regard that inhibits all things patient safety and quality in healthcare practice. For example, no current literature (including Darling [18] or Darwin [17]) identify any patient safety, clinical governance characteristics, or personal qualities and functions of the mentor in healthcare. This, therefore, may inhibit the universal and international appeal of the term “mentor”. When focusing on patient safety and clinical governance functions of the role in wider contemporary healthcare practice, a unique and evolving relationship is presented.

Despite the importance placed upon the role and responsibilities of the mentor for facilitating mentee learning, a continually perceived decline in the quality and standards of nursing care and services in some areas as opposed to others is witnessed. This could be attributed to the fact that there is limited emphasis placed upon the mentor for raising awareness and the application of clinical governance with their mentee’s in clinical practice. Whilst there is little doubt that mentorship will contribute positively to personal growth and development, there has been scant attention to the impact it has upon successful learning and healthcare practice. Success is largely assumed rather than demonstrated. Huybrecht et al. [23], Gopee [24], Eby et al. [27], Paglis et al. [29], Tonidandel, Avery and Phillips [30], and more recently Williams et al. [31] and Gray and Brown [32], indicate that there is little evidence to suggest that mentoring adds value to outcomes or indeed to improving quality and safety through clinical governance. However, Snowden and Hardy [26] and Snowden [28] allude to the potential benefits of mentorship on academic performance and learning for mentees and mentors in health and social welfare, suggesting that mentorship can enhance performance. This research study explores the potential relationship between mentorship, health, social care practice, and the association with clinical governance.

Darling [18] continues to be cited as “seminal” work when researchers explore the characteristics of the mentor. However, it is widely accepted that there has been a significant shift in the socio-cultural dimension of practice during the past 30 years. This research project explores those dimensions reported by Darling and assesses their relevance to contemporary health and social care practice. The research also provides the opportunity to assess the influence of Darwin [17] dimensions of mentoring on healthcare mentorship practice and how this may influence safety, quality, and clinical governance in the future. The challenge is in identifying the possible relationship between the mentor and mentee and its impact on raising awareness of clinical governance in practice.

### 1.2. Aims

The research aimed to explore primary healthcare workers and their mentor’s awareness of mentorship and clinical governance as part of a local Continuing Professional Development (CPD) program. To establish if any relationship between the mentors, the student as a mentee, and their awareness and application of clinical governance in nursing practice exists.

The aim was realized through a series of objectives as follows:

(1) To identify the key characteristics of the mentor from the perspective of the mentee and mentor;
(2) To investigate primary healthcare workers awareness of mentorship and clinical governance;
(3) To critically explore the factors that influence mentorship relationship and the application of clinical governance within primary healthcare practice;
(4) To identify the role mentorship plays in facilitating clinical governance in primary healthcare practice;
(5) To establish the role of mentoring and clinical governance in the delivery of CPD of primary healthcare workers.

## 2. Methodology

A quantitative research design was adopted where the researchers developed and integrated previously validated questionnaires. This incorporated Darling’s Measuring Mentor Potential Scale [18], Darwin’s Dimensions of Mentoring [17], and the Clinical Governance Awareness Questionnaire [5]). The newly devised questionnaire called “Mentorship and Clinical Governance Awareness” is presented in Questionnaire S1 (Supplementary Questionnaire S1).

### 2.1. Data Collection

Robson and McCartan [33] assert that triangulation in data collection is an essential tool in real world enquiry to promote rigor, advocating that similar patterns of findings from different data gathering tools increases confidence and trustworthiness in their validity. By seeking clarification through a range of data sources, Robson and McCartan [33] infer that the validity of a study, “the case”, is increased. The data sources in this study complemented each other and enabled the collection of rich data for analysis to occur.

### 2.2. Instrument

Following permissions, the newly designed and previously validated postal questionnaires, incorporating Darling’s Measuring Mentor Potential scale [18], Darwin’s Dimensions of Mentoring [17], and the Clinical Governance Awareness Questionnaire [5] were used to obtain the following information: The demographic characteristics, level of education, practice knowledge; investment of time, perceived drawbacks, and benefits; skills, qualities, duties, attitudes, characteristics, and competencies of the mentors and mentees. The questionnaire was also used to collect data on attitudes, characteristics, and competencies; patterns of use of the mentoring scheme; support offered and gained; mentor perceptions of any advantages and disadvantages of participating in this scheme alongside their awareness and understanding of clinical governance. The surveys contained a mixture of fixed responses on a five-point Likert Scales and some open-ended questions within the clinical governance component of the questionnaire.

### 2.3. Sample

Inclusion criteria consisted of a convenience sample of 480 primary healthcare workers registered on a Continuing Professional Development Program undertaking either the Certificate in Higher Education and/or Certificate in Higher Education in Primary Care. Those who were not registered on the above courses were excluded. The rationale for this was the fact that those attending the courses were either mentees and or mentors.

The Continuing Professional Development Program cohorts occurred in July, September, and October amounting to six programs. Each program catered for a minimum of 20 and to a maximum of 40, given a potential recruitment and sample size of 120 minimum and 240 maximum, and a corresponding number of invited mentors. The total recruitment and sample size could potentially be 240 minimum and 480 maximum.

## 3. Results

The data analysis was undertaken combining descriptive and inferential statistics through the application of Statistical Package for Social Science (SPSS) Version 21 [34].

A total of 112 completed questionnaires were included for the analysis amounting to a 23% response rate. This, according to Timmins, McCabe, and McSherry [35] is a reasonable response (> than 20%) for this type of postal questionnaire. The breakdown by population for primary healthcare workers was as follows: Registered nurses n = 39/35%, Midwives n = 3/2%, Healthcare Assistants n = 68/61%, and other (non-disclosed title) n = 2/2%. Grade (pay and recognition of experience) ranged from bands 2–9 (the higher the band equates to more pay and recognition). Hours worked was 25–30 hours n = 25/22% and full-time n = 42/38%. A total of 67/60% were mentees and 39/35% were mentors. Range of years working in the National Health Service was from 1–37 years with a mean average of 14 years.

### 3.1. Findings from the Mentorship and Clinical Governance Awareness Questionnaire

The numerical findings from Part A—Characteristics of a Mentor are detailed in Table 1 below.

The frequency values/percentage findings from Part B—Personality characteristics of a mentor are detailed in Table 2 below.

The frequency values/percentage findings from Part C—Clinical Governance Awareness Questionnaire are detailed in Table 3 below.

### 3.2. Justification for Factors Analysis

The justification for adopting a principal component factor analysis using statistical package for social sciences (SPSS version 21) [34] as opposed to exploratory factor analysis [33,36] was primarily due to the fact that a number of pre-defined ideas and potential dimensions were identified in the set of variables [36]. Consequently, this reduced the large number of variables contained within the three parts of the questionnaire previously identified in the instrument section. Principal component factor analysis facilitated this by combining part 1—Darling [18] characteristics of an effective mentor and part 2—Darwin [17] personality characteristics of an effective mentor. Following varimax rotation, the data extraction enabled four new factors to be identified having eigenvalues of one. These factors were labeled 1–4 which also identified four primary characteristics of a mentor outlined in Table 4 below.

The limitation of adopting factor analysis in this context was as follows. The interpretation of the data and naming of the variables was difficult where there were lots of correlations, for example, in factor 1. Factor analysis is only as good as the researcher’s experiences and ability to enter data accurately and completely. An incomplete data set may culminate in inaccurate results and interpretations.

### 3.3. Mentorship and Clinical Governance

The results from Table 3 illustrated that the majority of participants overwhelmingly agreed with question 12. They felt that their understanding and application of clinical governance principles is enhanced by the role of the mentor. Despite this finding, the data from the clinical governance awareness questionnaire revealed a slight contradiction by the fact that in response to question 13, only n = 30/27% had witnessed change directly from engaging with clinical governance principles and processes. Similarly, in response to questions 19 (main drivers for mentorship) and 20 (barriers affecting engagement in clinical governance activities), participants offered limited evidence in response to these key influencers for quality improvement and patient safety.

## 4. Discussion

The completion rate of 23% is considered to be valid in a survey questionnaire of this type, Timmins, McCabe, and McSherry [35] further corroborated by Water et al. [37] who also assert the importance of using pre-validated instruments in order to obtain reliable results, hence all questionnaires used were pre-validated and tested.

Table 1 illustrates that there are four key characteristics of Darling’s [18] original nine that continue to remain relevant and are deemed essential in enabling a mentor to be effective in contemporary practice. These are as follows.

*Teacher-Coach* was identified to be the number one characteristic of an effective mentor according to 105 respondents. This could be regarded as an individual who provided guidance on clinical problems and who taught and explained how to prioritize and develop interpersonal skills.

*Feedback-Giver* was identified by 100 respondents as the number two priority. This could be regarded has someone who provided a lot of positive, constructive feedback identified when practice was not right and how to improve practice by examining it.

*Standard-Prodder* was considered by 94 respondents to be the third key characteristic of an effective mentor. This is described as someone who is very clear about what was required, helped drive towards high aspirations and standards and would keep prodding.

*Eye Opener* was the fourth characteristic that impacts upon the mentoring role according to 85 individuals. This is portrayed as a mentor who “opened eyes”, encouraged interest in research, and helped to understand the nature and “politics” of the professional and healthcare environment.

The above findings identifying “feedback giver” as a characteristic, was not surprising due in part to its frequency of occurrence in definitions [26]. The significant finding is that “teacher-coach”, “standard-prodder”, and “eye opener” take prominence over characteristics such as “idea bouncer”, “problem solver”, and “challenger.” The former characteristics have all historically been strongly highlighted as effective characteristics of an effective mentor [38]. These changes in perceptions could be attributable to the challenges facing individuals working in healthcare organizational cultures and environment that are complex, dynamic, and clinical governance and patient safety focused [5]. The emphasis is placed on forward facing and the here and now activities to the jeopardy of career and continuous professional development.

Whilst mentorship has been introduced in nursing to develop skills and competence [39] of students and newly qualified practitioners, it continues to be regarded by many to be a vital strategy in the development of healthcare education [22,24,25,27,40]. Furthermore, reflection and the acquisition of reflexive skills are associated with the role of the mentor and indeed critical practitioner. It are these very practices of the “challenger” who encourages self-reflection by looking back at decisions and encouraging justification of those care decisions, by adopting a solution-focused approach to solving problems presented in a relationship. The notion of “risk aversion” seems to be taking precedence where the mentor does not have the time and security to engage with the mentee to be encouraged to explore ideas, issues, and “bounce ideas” regarding situations, incidents, and events in practice. Whereas the findings demonstrate that mentees were looking for their mentors to have very clear aspirations of the expected standard of care and to provide clear guidance on clinical problems and how to prioritize care. However, these characteristics seem to be placed in lesser importance with the mentor who is focusing on clinical governance and patient safety issues.

With regard to Table 2 drawing upon Darwin’s [17] personality characteristics, further significant findings are illustrated. Firstly, there are five key personality qualities desired in the mentor that contributes to successful mentorship: Trust, approachability, non-bias and non-judgmental, empathy, friendliness, and willingness. Significantly, these are all potential attributes akin to sound clinical governance and “duty of candor” attributed by the Francis Public Inquiry [11]. The finding is again demonstrating a focus on “risk aversion” and “reactive” approach to care. 

Trust, approachability, friendliness, empathy, and the ability to be non-judgmental are fundamental features to enable the mentee to reflect and share practice. The mentee, in order to develop excellence in practice, must be able to freely, without fear of penalty, share incidents when practice was not right. This approach enables the mentor to utilize their “inside knowledge” [40] and expertise to improve practice by examining it and providing guidance that can be used to enhance practice. This process of reflection must, as Ruth-Sahd [41] and Moon [42] assert, be based on honesty and openness.

The importance of willingness to perform the role of the mentor is well documented [23,40,42,43], and as reported by Eby et al. [27], failure of the mentor to support the mentee and neglect of the mentor are two of the most commonly reported problems reported within mentoring programs. Clearly, coercion or those potential mentors who are reluctant to participate in the role should be avoided by mentoring program leaders and institutions [31]. Whilst the role of the mentor is difficult to define due to its multi-faceted nature, reflecting the diverse contexts where mentoring takes place [2], there is some agreement upon what personality characteristics and features support the development of the mentoring relationship. Honesty, willingness, approachability, empathy, and friendliness form the basis of successful mentoring outcomes, [24,40,41,42,43,44,45].

Table 3 highlights a sound understanding and awareness of the principles and practices aligned to clinical governance. The majority of respondents recognize that clinical governance plays a major part in improving patient care and is an integral part of their role and responsibility. They recognize the benefits of clinical governance to oneself, their team, organization, and wider patient care. Interestingly and worryingly, despite the wider value of clinical governance frameworks and their association to patient safety and quality, the majority of respondents indicated there was inadequate peer and organization support along with education and training opportunities to improve knowledge, understanding, and competence in this important area. The culmination of this for both mentees and mentors is a lack of confidence to engage with these activities in practice. Significantly, the results indicate that clinical governance activities can be enhanced by the mentor. This could possibly be the first study to recognize this in practice.

**Figure 1:**
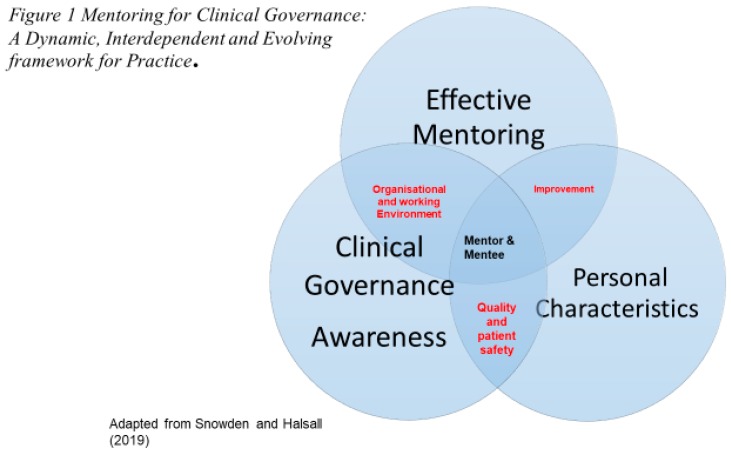
Reveals a new emerging framework termed “Mentoring for Clinical Governance: A Dynamic, Interdependent, and Evolving Framework for Practice”. This new framework facilitating mentorship was identified following a synthesis of all of the quantitative data.

A synthesis of the findings identified in Table 1, Table 2, and Table 3 reveal a new emerging conceptual framework for integrating clinical governance within a mentorship role and responsibilities in the future. This is because the utilization of Darling’s [18] key characteristics of a mentor, Darwin’s [17] personal traits of a mentor combined with McSherry and Pearce’s [19,46] clinical governance awareness, offer new insights and understanding of mentoring in modern, dynamic, complex systems of healthcare and the impact on patient safety and clinical governance systems in healthcare practice. An effective mentor in healthcare requires a consolidation and combination of Darling [18], Darwin [17], and McSherry and Pearce [19,46] characteristics in the future. To achieve this, the research findings suggest that an alternative or adoption to existing mentoring frameworks and approaches be undertaken. These newly identified interdependent elements are identified in Figure 1 below.

Mentors and mentees awareness of clinical governance and its importance to improving patient safety, quality, and the delivery of compassionate care and services should focus on three key factors. These are as follows:

*“Effective Mentoring”* is associated with enhancing an individual’s mentorship characteristics of “Teacher-Coach”, “Feedback-Giver”, “Standard-Prodder”, and “Eye Opener”. This is essentially a mentor who provides mentee guidance on clinical problems, and when teaching in clinical practice, explains how to prioritize and develop interpersonal skills with their mentee. They focus *on* providing positive, constructive feedback identified when practice was not right and how to improve practice by examining it in partnership with their mentee. Furthermore, they are someone who is very clear about what was required from their mentee, helped drive towards high aspirations and standards by constantly prodding and rewarding their mentee towards successful completion of their work. This is a mentor who “opens eyes” by encouraging interest in research and helping mentees to understand that the nature and “politics” of the professional and healthcare environment is constantly evolving and changing.

*“Personal Characteristics”*, five key individual mentor and mentee personality qualities are deemed essential to be a successful mentor. These are: Trust, approachability, being non-bias and non-judgmental, empathy, friendliness, and willingness to support. These personal characteristics are important when focusing on patient safety and clinical governance related issues because they resonate with the core principals of honesty, openness, trust, transparency, and probity. The mentorship role and responsibilities should focus on harnessing and strengthening these with the mentor and mentee relationship.

*“Clinical governance awareness”* is about education and training where both the mentor and mentee know about what clinical governance is and is not. Within the proposed mentorship framework, clinical governance is about encouraging individuals to learn and share from their experiences both positive and negative in an open and transparent way. It is about having an appreciation of how the organizational working environment and workplace culture can influence the delivery and outcomes of safe, compassionate quality care both positively and negatively. It is about knowing how the roles and responsibilities of both the mentor and mentee can be improved along with the clinical learning and teaching environment in which they work. Clinical governance is about having the knowledge, understanding, skills, support, and confidence to challenge poor practice, to reward and celebrate excellence in practice whilst respecting the individual’s dignity at all times. It is about putting the person at the center stage.

## 5. Conclusions

Exploring the role of mentoring in facilitating patient safety and clinical governance within primary healthcare offers new and original insights for all aspects of health and care. The research offers original insights into the role of the mentor in facilitating safe and effective primary healthcare practice with their mentees. The findings provide the emergence of a new way of facilitating patient safety and clinical governance through the “Mentoring for Clinical Governance: A Dynamic, Interdependent and Evolving Framework for Practice” model. This model supports the recommendations of the World Health Organisation^46^ that health professions must improve support structures in order to enhance expertise.

The authors acknowledge a limitation of the research, which lies in the fact that the sample was primarily from primary healthcare. However, the findings have global appeal and may be useful in developing this important aspect of facilitating patient safety, governance through mentorship in the following reasons. Firstly, it highlights the importance of the mentor’s role and responsibilities in both recognizing and facilitating patient safety, quality improvement for both themselves and their mentee(s). Secondly, the questionnaire confirms both the key characteristics of mentoring and individual characteristics of the mentor deemed essential to create an appropriate clinical learning and working environment in which clinical governance nurtured. Thirdly, the findings demonstrate three factors that collectively inform the development of the new “Mentoring for Clinical Governance: A Dynamic, Interdependent, and Evolving Framework for Practice”. Fourthly, the findings show the importance of the mentor role in facilitating patient safety and clinical governance within primary healthcare practice. Finally, mentoring for clinical governance requires a review of existing mentoring education programs and training to see how these new findings can be incorporated into existing continuing professional development education programs in the future.

## 6. Recommendations

There are several major recommendations from the research findings, such as:

- Using the newly devised and validated questionnaire to be applied globally across the health and care settings for a larger scale research;
- To further review the key characteristics of mentoring and individual characteristics of the mentor in facilitating clinical governance and patient safety within the clinical learning environment for the mentees through qualitative methodologies;
- To encourage Higher Education Institutions (HEIs) and Nursing Colleges to review existing mentor programs to incorporate ways of highlighting the importance of the mentor in facilitating patient safety and clinical governance within clinical health and care programs.

## Figures and Tables

**Table 1:** Characteristics of a mentor.

Characteristic	1	2	3	4	5
	Not Relevant/Important	Less Relevant/Important	Neutral	Relevant/Important	Very Relevant/Important
**1**	1/0.9%	1/0.9%	16/14%	35/31%	59/53%
**2**	1/0.9%	0	6/5%	38/34%	66/59%
**3**	1/0.9%	3/3%	8/7%	33/29%	67/60%
**4**	1/0.9%	5/4%	21/19%	38/34%	47/42%
**5**	1/0.9%	4/4%	26/23%	49/44%	32/29%
**6**	1/0.9%	4/4%	22/20%	42/37%	43/38%
**7**	2/2%	2/2%	23/20%	47/42%	38/34%
**8**	6/5%	17/15%	31/28%	30/27%	28/25%
**9**	1/0.9%	9/8%	22/20%	48/43%	32/29%

**Table 2:** Personality characteristics of a mentor.

Characteristic	1	2	3	4	5
	Not Relevant/Important	Less Relevant/Important	Neutral	Relevant/Important	Very Relevant/Important
**1**	1/0.9%	1.9%	7/6%	38/34%	65/58%
**2**	1/0.9%	3/3%	10/9%	49/44%	49/44%
**3**	1/0.9%	4/4%	30/27%	49/44%	28/25%
**4**	1/0.9%	2/2%	11/10%	40/36%	58/56%
**5**	2/2%	2/2%	17/15%	43/38%	4/43%
**6**	2/2%	1/0.9%	14/12%	46/41%	49/44%
**7**	1/0.9%	2/2%	29/26%	49/44%	31/28%
**8**	1/0.9%	0	6/5%	38/34%	67/60%
**9**	1/0.9%	1/0.9%	13/12%	35/31%	62/55%
**10**	1/0.9%	1/0.9%	17/15%	37/33%	56/50%
**11**	1/0.9%	3/3%	16/14%	55/49%	37/33%
**12**	1/0.9%	0	12/11%	58/52%	41/37%
**13**	1/0.9%	2/2%	12/11%	51/45%	46/41%
**14**	2/2%	1/0.9%	13/12%	62/55%	34/30%
**15**	2/2%	2/2%	12/11%	57/51%	39/35%
**16**	3/3%	1/0.9%	10/9%	54/48%	44/39%

**Table 3:** Clinical Governance Awareness Questionnaire.

Question	1	2	3	4	5
	Strongly Disagree	Disagree	Neutral	Agree	Strongly Agree
**1**	2/2%	4/4%	9/8%	52/46%	45/40%
**2**	3/3%	2/2%	15/13%	50/45%	42/37%
**3**	2/2%	3/3%	22/20%	54/48%	31/28%
**4**	1/0.9%	3/3%	19/17%	62/55%	27/24%
**5**	1/0.9%	3/3%	27/24%	47/42%	34/30%
**6**	2/2%	4/4%	31/28%	53/47%	22/20%
**7**	2/2%	7/6%	37/33%	39/35%	27/24%
**8**	5/4%	13/12%	35/31%	38/34%	21/19%
**9**	1/0.9%	8/7%	41/37%	31/28%	31/28%
**10**	1/0.9%	10/9%	30/27%	42/37%	28/25%
**11**	2/2%	7/6%	33/29%	51/45%	19/17%
**12**	2/2%	8/7%	20/18%	54/48%	28/25%
	Yes			No	
**13**	30/27%			77/69%	
**14**	40/36%			70/62%	
**15**	51/45%			59/53%	
**16** *	39/35%			73/65%	
**17** *	32/29%			80/71%	
**18** *	26/23%			86/77%	
**19** *	25/22%			87/78%	
**20** *	33/29%			79/70%	
**21** *	30/27%			81/72%	
	1	2	3	4	
**22**	8/7%	94/84%	9/8%	0	
**23**	1/9%	103/92%	6/5%	0	

* Questions 16–21 were qualitative in nature, and where a response was made, it was indicated by a yes and/or no answer. A summary of the qualitative data was undertaken but is not included for the purpose of this article.

**Table 4:** Emerging characteristics and labeling of factors following principal component.

Factor 1	Factor 2	Factor 3	Factor 4
Characteristics 1: A Facilitatory Adviser	Characteristic 2: Critically Enabling Facilitator	Characteristic 3: A Change Facilitator	Characteristic 4: An Approachable Facilitator
Darling (1984) [18]			
Feedback-Giver 0.614	Standard Prodder 0.611	Standard Prodder 0.534	
	Teacher-Coach 0.518		
	Eye Opener 0.636		
	Door Opener 0.646	Career Counsellor 0.659	
	Idea-Bouncer 0.552		
	Problem-Solver 0.550		
Darwin (2004) [17]			
Approachable 0.775			Friendly 0.535
Friendly 0.623			
No bias and non-judgmental 0.778			
Patient 0.797			
Enthusiastic 0.792			
Negotiation Skills 0.728			Nice 0.709
Trust 0.700			
Communication 0.748			
Empathy 0.747			
Empowering 0.790			
Confident 0.794			
Motivation 0.842			
Reflective 0.783			
Leadership 0.800			
Willingness 0.758			

## Supplementary Materials

The following are available online at https://www.mdpi.com/2227-9032/7/4/113/s1, Questionnaire S1: Mentorship and Clinical Governance Awareness Questionnaire.
