# Supplementary material for: Exploring Primary Healthcare Students and Their Mentors’ Awareness of Mentorship and Clinical Governance as Part of a Local Continuing Professional Development (CPD) Program: Findings of a Quantitative Survey
Source: Healthcare (Basel). 2019 Oct 2;7(4):113. doi: 10.3390/healthcare7040113 (PMC6955968; doi:10.3390/healthcare7040113)
Supplement: Supplementary file 1 [file healthcare-07-00113-s001.pdf]

Supplementary: Questionnaire

Mentorship and Clinical Governance Awareness Questionnaire

There are four parts to this questionnaire, please complete all sections:

Parts A and B is concerned with collecting information on those physical and personality characteristics of mentorship and the factors that may influence the mentoring relationship: Part C is about your awareness of clinical governance and part D associated with demographic information.

PART A *Adapted from the works of Darling (1984) with kind permission for reproduction by Journal of Administration in Nursing

(Use of Rating Scale 1-5) and opportunity to comment

The following section of the questionnaire presents a number of characteristics, identified by health care practitioners as significant in their guidance and growth. Using the scale of 1 – 5 (where 5 is very relevant/important and 1 is of lower relevance/importance) use this questionnaire to assess the relevance and importance your perceptions of the mentor's, an explanation is given for each characteristic where appropriate. Please √ and rate your response as 1 if not very relevant/important and 5 if very relevant/important.

	1	2	3	4	5
**Standard-prodder**<br>Some one who was very clear about what was required, helped drive towards high aspirations and standards and would keep prodding.	❑	❑	❑	❑	❑
**Teacher-Coach**<br>Someone who provided guidance on clinical problems, taught and explained how to prioritize and develop interpersonal skills.	❑	❑	❑	❑	❑
**Feedback-Giver**<br>Some one who provided a lot of positive, constructive feedback, identified when practice	❑	❑	❑	❑	❑

wasn’t right and how to improve practice by examining it.					
**Eye Opener**<br><br>Someone who “opened eyes” encouraged interest in research and helped me to understand the nature and “politics” of the professional and health care environment	❑	❑	❑	❑	❑
**Door-Opener**<br><br>Someone who made services and opportunities available and adopted an inclusive approach.	❑	❑	❑	❑	❑
**Idea-Bouncer**<br><br>Encourages the mentee to explore ideas, discuss issues, problems, and goals and bring things into focus	❑	❑	❑	❑	❑
**Problem-Solver**<br><br>Someone who adopted solution focus and problem solving approaches	❑	❑	❑	❑	❑
**Career Counsellor**<br><br>Someone who encourages personal development and career planning. Looking to the future	❑	❑	❑	❑	❑
**Challenger**<br><br>Someone who encouraged self reflection, looking at decisions and encouraging justification for the point made	❑	❑	❑	❑	❑

PART B (use of Rating Scale 1-5)

The following section asks you to consider a number of personality characteristics, identified by health care practitioners as significant to the mentor personality. Using the scale of 1 – 5 (where 5 is very relevant/important and 1 is of lower relevance/importance). Please √ the appropriate box.

	1	2	3	4	5
• Approachable	❑	❑	❑	❑	❑

• Friendly	❑	❑	❑	❑	❑
• Nice	❑	❑	❑	❑	❑
• Non bias and non-judgmental	❑	❑	❑	❑	❑
• Patient	❑	❑	❑	❑	❑
• Enthusiastic	❑	❑	❑	❑	❑
• Negotiation Skills	❑	❑	❑	❑	❑
• Trust	❑	❑	❑	❑	❑
• Communication	❑	❑	❑	❑	❑
• Empathy	❑	❑	❑	❑	❑
• Empowering	❑	❑	❑	❑	❑
• Confident	❑	❑	❑	❑	❑
• Motivation	❑	❑	❑	❑	❑
• Reflective	❑	❑	❑	❑	❑
• Leadership	❑	❑	❑	❑	❑
• Willingness	❑	❑	❑	❑	❑

PART C: CLINICAL GOVERNANCE AWARENESS QUESTIONNAIRE (McSherry and Pearce 2010) - Reproduced with kind permission from Wiley, Blackwell Publishers.

In relation to the following questions, on a scale of one to five, one being strongly agree, and five strongly disagree, please ◎ tick the box that matches your view most closely.

	1	2	3	4	5
Clinical governance has a large part to play in improving patient care.	❑	❑	❑	❑	❑
Clinical governance is part of my role and responsibility.	❑	❑	❑	❑	❑
In using the clinical governance framework to support my practice I see benefit for myself.	❑	❑	❑	❑	❑
Engaging with the clinical governance framework can influence patient care.	❑	❑	❑	❑	❑
Clinical governance is a useful framework to change clinical practice at an individual, team and organisational level.	❑	❑	❑	❑	❑
Engaging with the clinical governance framework can have a benefit in changing culture in the working environment.	❑	❑	❑	❑	❑
I have sufficient support and encouragement from peers and professionals to engage with the clinical governance framework.	❑	❑	❑	❑	❑
I have sufficient support from the management within my Directorate / clinical area to engage with clinical governance framework.	❑	❑	❑	❑	❑
In my training I received adequate information about what the clinical governance framework is and how it may aid improve quality services.	❑	❑	❑	❑	❑
I have a basic knowledge and understanding of clinical governance and associated systems and processes.	❑	❑	❑	❑	❑
I am confident to engage with the clinical governance framework.	❑	❑	❑	❑	❑
Is clinical governance enhanced by mentorship	❑	❑	❑	❑	❑

Have you witnessed any change in practice that was related from individuals, teams or organisation engaging in the clinical governance framework?	Yes ❑	No ❑		If you answered yes to the Questions please explain opposite
Have you read any professional journal articles or books on clinical governance or mentorship?	Yes ❑	No ❑		If you answered yes to the Questions please explain opposite
Have you had any clinical governance education and training?	Yes ❑	No ❑		If you answered yes to the Questions please explain opposite

Please write your response in the box provided opposite.	
What do you feel are the main drivers for introducing clinical governance into the healthcare service?	
What do you understand by the term clinical governance?	
What would you describe as the key components of clinical governance?	
What do you feel are the main drivers for introducing mentorship into the healthcare service?	

Can you list the three greatest barriers affecting your engagement with the clinical governance frameworks ?	**1.**<br><br>**2.**<br><br>**3.**
Can you list the three greatest barriers affecting your engagement with the Mentorship in practice ?	**1.**<br><br>**2.**<br><br>**3.**

Which of the following statements best describes clinical governance to you (✓ the appropriate box)

Clinical governance is the role and responsibility of chief executives and managers in maintaining quality care and services	❑
Clinical governance is a quality framework and should be part of everyone's role and responsibility	❑
Don't know	❑
None of the above	❑

Clinical governance is only about dealing with risks and managing complaints	❑

For clinical governance to occur in an organisation the following systems and processes need to be in place:<br><br>• Risk management<br>• Performance management<br>• Quality improvement programme<br>• Information<br>• Accountability	❑
Don’t know	❑
None of the above	

Please provide any necessary information relating to your answers or regarding to the questions contained in the questionnaire in the box provided.

Please identify the question/s your comments relate to

Part D: Demographic Information

What is your position.

Doctor	❑
Hospital Midwife	❑
Community Midwife	❑
Registered Nurse	❑
**Allied Health Professional:**<br><br>Please state your profession opposite	
Health Care Assistant	❑

When did you qualify as a Healthcare Professional

Please state your grade:

Please state which clinical specialty you currently work in:

Which of the following hours do you work?	
10-14	❑
15-20	❑
21-24	❑
25-30	❑
31-37.5	❑

Please state your professional qualifications and additional qualifications.
**Professional:**<br><br><br>**Additional:**

Thank you very much
for taking the time to complete and return this questionnaire.

All your responses will be confidential and no comment will attributable to you
